# The potential of neuroscience for health sciences education: towards convergence of evidence and resisting seductive allure

**DOI:** 10.1007/s10459-016-9733-2

**Published:** 2016-11-07

**Authors:** Anique B. H. de Bruin

**Affiliations:** School of Health Professions Education, Maastricht University, P.O. Box 616, 6200 MD Maastricht, Netherlands

**Keywords:** Educational Neuroscience, Evidence, Theory building, Translation to education, Convergence of evidence, Seductive allure

## Abstract

Since emergence of the field ‘Educational Neuroscience’ (EN) in the late nineties of the previous century, a debate has emerged about the potential this field holds to influence teaching and learning in the classroom. By now, most agree that the original claims promising direct translations to teaching and learning were too strong. I argue here that research questions in (health professions) education require multi-methodological approaches, including neuroscience, while carefully weighing what (combination of) approaches are most suitable given a research question. Only through a multi-methodological approach will convergence of evidence emerge, which is so desperately needed for improving teaching and learning in the classroom. However, both researchers and teachers should become aware of the so-called ‘seductive allure’ of EN; that is, the demonstrable physical location and apparent objectivity of the measurements can be interpreted as yielding more powerful evidence and warranting stronger conclusions than, e.g., behavioral experiments, where in fact oftentimes the reverse is the case. I conclude that our tendency as researchers to commit ourselves to one methodological approach and to addressing educational research questions from a single methodological perspective is limiting progress in educational science and in translation to education.

The subdiscipline of Educational Neuroscience (EN) has a history of assertions about the potential of brain insights to transform classroom teaching and learning (e.g., Ansari 2005; Ansari et al. 2011; Blakemore and Frith 2005). Dehaene (2011) takes this one step further contending that in-depth knowledge of the workings of the brain will “spontaneously” lead teachers to improve their teaching methods. After two decades of EN research (Bruer 1997), however, no convincing evidence of direct or spontaneous improvement of teaching and learning as a result of neuroscience insights exists (Bowers 2016). Several neuroscientific findings are mere brain-based replications of established phenomena from behavioral research (e.g., Carew and Magsamen 2010), and as such do not contribute to progress in education. As Roediger et al. (2012) put it, neural processes underlie every cognitive process, and establishing where these processes take place in the brain does not necessarily add to explaining the nature of these processes, let alone to formulating design principles for training.

By now, the strong claim that EN should be able to directly improve teaching in the classroom receives virtually no support (Gabrieli 2016). It is time to let go of the “brain scan to lesson plan” credo (Blakemore and Frith 2005) for several reasons. The limitations of EN methods only allow well-controlled learning tasks, reducing generalizability and ecological validity. EN typically examines phenomena that have been studied in a robust manner in behavioral experiments, but lacks the flexibility and possibilities for contextual variation that characterize the latter and that are needed to come to design of training principles. Critics of EN are eager to confirm that the value of EN is negligible or, more coarsely, that it simply will never work (Bowers 2016).

## Convergence of evidence

But dismissing a scientific (sub)discipline based on the methods it identifies with, is one bridge too far. The direct linkage of a discipline with methods contributes to the rough water educational science is in, but I will return to that later. In essence, EN is a basic neuroscience that relates to educational theory (Willingham and Lloyd 2007) and that has as its object the characteristics and development of the learning brain, or context factors that relate to the learning brain (e.g., work on teachers’ neuromyths, (Dubinsky et al. 2013). As such, EN makes perfectly valid contributions to science, for example by confirming insights previously gained in behavioral research (such as in the article by Rourke et al. this issue) or by exposing discrepancies between behavioral and neuroscience findings (such as in ERP research on early and advanced readers showing that automation of letter-sound correspondence takes much longer than behavioral studies suggest; (Froyen et al. 2009). That is, EN provides insight into how the brain changes because of learning or how the brain is active during learning. Just as we do not value social neuroscience based on its impact on societal happiness, we should not value EN on its ability to reform education (Gabrieli 2016).

Moreover, let us not fall prey to maintaining double standards; The bold claims EN is explicitly held accountable for are similar to what is argued in several other domains. Expertise researchers, for example, often defend their research approach stating that knowledge about expert performance informs us about how to instruct novices (Ericsson et al. 2006). However, insight into how an expert solves a domain-specific problem does not directly tell us anything about how to teach novices. One could even question whether there is reason to assume that it will do so indirectly. Yet, a fierce debate regarding the claims on education that warrant existence of expertise research has never occurred, nor am I convinced that it will ever occur. And as long as we do not know where discoveries that will revolutionize education will originate from, it is worth spreading our money over several horses, instead of disqualifying one of the horses in advance.

If we agree that the ultimate goal of educational research is to improve teaching and learning in the classroom (where ‘classroom’ should be read as any environment were teaching and/or learning directly takes place), then we should also agree that the applied nature of this research field asks for a pragmatic approach when it comes to choosing and designing the methods to study any given research question within this field. That is, the research question should drive the choice of methods in such a way that chances for finding useful answers are optimized (Johnson and Onwuegbuzie 2004). However, in doing so, we should always endorse high standards of rigour (e.g., incorporate empirical performance measures, aim for reproducible results within and between learners, and elaborate theoretical models that hold after experimental validation, Ericsson et al. 2006). Yet, we must also acknowledge that description of design guidelines for training should only take place when converging evidence is obtained across multiple rigorous studies. This is not an issue that is specific to EN research. It applies to any methodological approach in educational research. Research using paired associates (studying word pairs, e.g., dog—chair) has led to robust findings and theoretical description related, for example, to the effect testing has on memory and learning (e.g., Roediger and Karpicke 2006a, b). Yet, all will agree that successful application to education is only possible after translation of these insights to context embedded research with authentic learning tasks [see e.g., work on the testing effect when learning clinical neurology (Larsen et al. 2013)]. Again, no need for double standards here. As Ringsted et al. (2011) explain, different methodological approaches in education have different aims. Where exploratory studies aim at theory testing and modelling, many experimental studies instead aim at justifying and some at clarifying. Translational studies, finally aim at implementing (see also Cook et al. 2008). The aims determine the type of additive value to science. In principle, EN or any other methodological approach can contribute to theory building and refinement.

The call for convergence of evidence should not be interpreted as a carte blanche for a ‘survival of the fittest’ approach in educational science. Design of research in educational science should be considered carefully and always abide by the guideline of maximum compatibility of research question and method. But having outlined how EN provides a valuable contribution to science in its own right, it is time, after two decades of EN research, to draw up the balance and outline what to consider when setting up neuroscience research in relation to educational questions. First, EN research is not suited for exploratory questions and should focus on robust phenomena (i.e., those that have replicable results on empirical performance measures). Given the wealth of data neuroscience research typically offers, an exploratory question will risk getting ‘lost in the brain’. This robustness will typically originate from behavioral research. If, at a behavioral level, we know little about the cause and consequences of a specific observed phenomenon, we should not expect neuroscience research to provide answers, for it will be impossible to design a study that can approximate the observed phenomenon and manipulate essential factors. For example, if we know next to nothing about how students approach multiple choice questions (MCQ) on anatomy exams, scanning students’ brains when answering MCQs on anatomy will only tell us that they were indeed using their brains while doing so. Second, it should be expected that the research question posed is in need of a brain-based answer. That is, contemplation should take place deliberating whether a neuroscience approach is in fact indispensable or a more cost effective behavioral approach could produce similar (or perhaps better) effects.

For those studies standing the test of these two recommendations, the question arises what type of EN research endeavors are particularly promising for health sciences education. If we look at the results of the two decades of research on EN, it is obvious that those relating to basic learning abilities such as reading and math have met with most success in terms of contributing to theory building. Especially those relating to early identification of at-risk children and predicting individual learning outcomes based on diverging patterns of brain activation hold promise (see Supekar et al. 2013 for a study in which neuroimaging measures correlated with learning from a tutoring program, where behavioral measures failed to do so). Unfortunately, this is not something that will prove particularly useful when studying first year medical students’ performance on, say, Physiology 101. In health sciences education, issues of learning disorders are of little importance and because basic abilities such as fluency of reading or math are hardly at stake, insights into how to teach these in a personalized manner will hardly resonate. On the other hand, fundamental questions regarding brain activation patterns of health sciences experts (compared to non-experts) during criterion tasks can provide meaningful contributions to expertise theory, whenever theory-based hypotheses relating to brain activation or brain areas can be formulated. Note, as mentioned above, that these studies will not necessarily lead to strategies for instruction of non-experts, at least not directly. Moreover, when cognitive processes are mostly implicit or too fast to unravel behaviorally, such as in pattern recognition (System 1 reasoning), neuroscience research can tap into the nature of these processes in a more fine grained manner and compare with neurocognitive processes similar to pattern recognition (e.g., object recognition, Wang et al. 2016). Note that these fast processes are typically observed in experts, and relate to components of expertise theory, adding to the conclusion that EN research in health sciences education appears to bear most value in relation to expertise-related questions.

## The seductive allure of Educational Neuroscience

Given the above, it is not surprising that the majority of research on EN in health sciences education investigates issues of relative expertise. That is, experts in a particular domain are compared to non-experts (mostly novices) on a criterion task (Ericsson et al. 2006). However, the fit of these criterion tasks for neuroscience research, despite their robustness in terms of replicable results, is often an issue of concern. Several fMRI studies have looked into clinical reasoning and have had to come up with ways to extrapolate fMRI paradigms on simple reasoning tasks, such as syllogistic reasoning (Goel 2007), to the complexity of diagnosing written clinical cases. This approach risks measuring apples as if they were oranges, because, in fact, we don’t know yet how to measure apples. One example arises when identifying the correct contrast as a baseline task for such a reasoning task. In the handful of studies that have been done (e.g., Durning et al. 2012), reading of an unrelated text is typically used, but the validity of this baseline task has not been examined separately. This is recognized as a potential drawback to the data analysis and results.

It is as if we are wanting too much, too fast. The ‘seductive allure’ of EN (Weisberg and colleagues (2008)) might be at play here. Weisberg et al. (2008) showed that results from neuroscience studies are often considered more informative than psychological studies, even when irrelevant neuroscience information is added to explanations of psychological phenomena. This seductive allure has an upside, e.g., as observed in the positive learning effect that followed after teaching children about neuroplasticity (Blackwell et al. 2007), but as with any seduction risks are involved too. First, there is the risk of throwing the baby out with the bath water. If we do not yet possess the knowledge to design valid fMRI research methods to answer educational questions (e.g., related to clinical reasoning), research based on those methods will be in vain. Second, even if we were able to design a valid paradigm, there is the risk of over- or misinterpreting results, leading to conclusions about educational theory and practice that are too strong. Several examples exist of fMRI studies describing direct implications for teaching and training, where translational research would be needed, first to small-scale classroom experiments, and then to a broader curriculum. Finally, the risk that I perceive as most ignored, but most detrimental to education is that of overlooking findings outside of EN that are just as, or even more valuable when it comes to explaining and improving students’ learning outcomes. Consider the two learning techniques that came out as most likely to improve learning outcomes in a review of 10 techniques that are frequently applied in education (Dunlosky et al. 2013): practice testing (or retrieval practice) and distributed practice. Practice testing is considered as any activity that requires learners to actively retrieve information from memory in response to a cue (e.g., a cue word or question). The positive effect of testing on memory and learning is usually termed the ‘testing effect’ or ‘test-enhanced learning’ (Larsen et al. 2008; Roediger and Karpicke 2006a, b). Distributing practice entails spacing learning over time, both within and across study sessions (instead of massing or ‘cramming’ all learning into a single session). This effect is typically called the ‘spacing effect’ (Dempster and Farris 1990).

Dunlosky and colleagues particularly looked into the effect of these techniques on long-term retention and their generalizability across learning conditions, student characteristics, materials, and criterion tasks. For both practice testing and distributed practice, utility was high. There was broad evidence of efficacy across student populations (from preschoolers to adults with Alzheimer’s disease; (Fritz et al. 2007; Tse et al. 2010) and in a wide range of tasks (from paired associates to surgery; (Barcroft 2007; Moulton et al. 2006). Steps that need to be taken to implement these techniques on a widespread basis in education are relatively small, and mostly concern designing and testing effective teacher training and examining the effect of the techniques at the curriculum level (Bjork et al. 2013; Dunlosky et al. 2013). Yet, we have little insight into the brain basis of the testing and spacing effect (Van Strien et al. 2007), and this is not holding back implementation in education in any way. It is simply not necessary and it is doubtful whether it will change conclusions already made based on behavioral research. The robustness of the effect as confirmed in cognitive and educational psychology research and the evidence for generalizability of the techniques across learning tasks and learner populations provides sufficient information to start designing education and teacher training. This applies not only to practice testing and distributing practice, but to several other learning techniques or phenomena that found a robust basis in cognitive and/or educational psychology research (e.g., the split attention effect, the modality effect; Mayer and Moreno 1998; Tabbers et al. 2004). Unfortunately, cognitive and educational psychology are less seductively alluring than neuroscience and, therefore, face more struggle to reach teachers and students. It is reassuring to observe the growing interest in, e.g., the testing effect in health sciences education (Larsen and Blair 2008). It shows that health sciences educators and researchers value amount of evidence for a phenomenon more than seductive allure of the evidence. Spreading of this interest to other robust learning phenomena from cognitive and/or educational psychology holds great promise for health sciences education.

## Conclusions

The linkage of education and neuroscience in a separately defined field has given rise to high expectations and polarized opinions. But it also represents a step back from a quest for convergence of evidence. Educational science is in need of (guidelines for) translational research, and could learn from medical research where the chain from preclinical to applied clinical research is well established, and where researchers embrace the importance of flexibility in methods and need for translational work. This is not to say we should become experts in several methodological streams, but we should be able to collaborate and communicate with experts in other streams maintaining an open attitude towards each stream, all in service of establishing the optimal research set up to answer our research questions. Only then will research make a valuable contribution to convergence of evidence in relation to specific phenomena or theories. Given the research evidence that we possess now, and in view of the questions that health sciences education is absorbed with, it is more likely that cognitive or educational psychology research will influence educational practice than will EN. But whenever EN research in health sciences education is employed to investigate a robust behavioral phenomenon and tests a theoretically driven brain-based hypothesis that cannot be answered in mere behavioral research, the findings will likely add to convergence of evidence surrounding that phenomenon, despite a lack of translation to educational practice. But let’s not judge EN on its direct contribution to educational practice nor push them towards untenable claims. If we wish for a revolution in educational science, we need all the help we can get.
